# Templated Twist Structure Liquid Crystals and Photonic Applications

**DOI:** 10.3390/polym14122455

**Published:** 2022-06-16

**Authors:** Yao Gao, Weiping Ding, Jiangang Lu

**Affiliations:** National Engineering Lab for TFT-LCD Materials and Technologies, School of Electronic Information and Electrical Engineering, Shanghai Jiao Tong University, Shanghai 200240, China; gaoyao123@sjtu.edu.cn (Y.G.); 20172240207@sjtu.edu.cn (W.D.)

**Keywords:** twist structure liquid crystals, templating technique, photonic properties, photonic applications

## Abstract

Twist structure liquid crystals (TSLCs) have attracted increasing attention in photonic applications due to their distinct properties: Bragg reflection, scattering, and optical rotation. However, there exist some issues due to the defects of TSLCs: weak thermal stability, narrow bandwidth, and complicated fabrication. In this review, we introduce the templating technique which includes device structure, templating process, and photonic properties of templated TSLCs to improve the issues. Furthermore, a variety of photonic applications including lasing, optical filters and gratings based on TSLCs with polymer templates are presented. Additionally, other applications of TSLCs are briefly introduced. Finally, the remaining challenges and future perspectives of templated TSLCs are proposed.

## 1. Introduction

**Figure 1:**
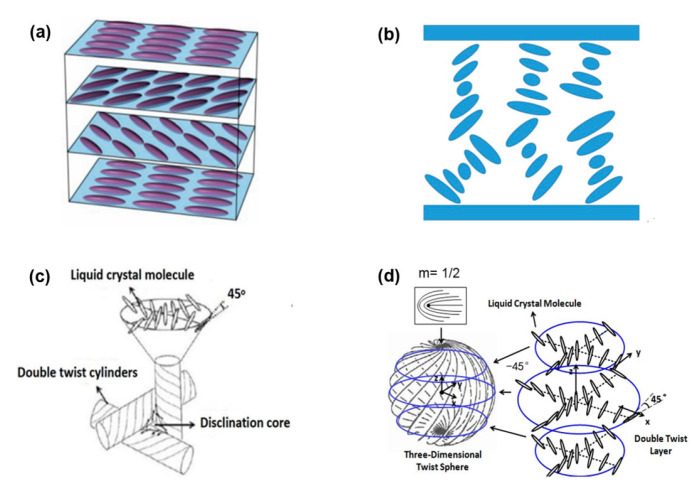
The arrangements of LC molecules in (**a**) planar texture state CLC; (**b**) focal conic state CLC; (**c**) BPLC; (**d**) SPLC.

Liquid crystal (LC) is a soft matter that combines crystalline-like solid ordering with fluid-like behavior. Twist structure (TS) LC is a class of variant LC that the directors of LC molecules are twisted to form the twist structure. TSLCs consist of cholesteric liquid crystal (CLC), blue phase liquid crystal (BPLC), and sphere phase liquid crystal (SPLC). The CLC, chiral nematic phase LC [1], has a self-assembly twist structure with the director exhibiting a helical form, which comes from the molecular chirality of rod-like molecules. If the structure was cut in a direction perpendicular to the helical axis, local nematic order would appear. A nematic LC (NLC) phase is an achiral phase that represents a purely orientational order of elongated molecules. Two commonly occurring in director axis alignments of CLCs are planar twist states and focal conic states, as shown in Figure 1a,b. In planar state CLCs, the cholesteric structure is often shown in a stack of layers with rod-like molecules. The LC molecules are parallel to each other within the layers and rotate by a certain twisted angle of each layer with respect to the neighbors. The focal conic state CLCs have a multi-domain structure with a disorder of molecule arrangement, which appears characteristic of random arrangement of helical axes in each domain. BPLCs appear as a regular array of double twist cylinders (DTCs), where the LC directors are twisted from −45° to +45° about any radius of the cylinder, separated by a network of disclination lines in a narrow temperature range between the isotropic phase and the chiral nematic phase [2,3,4,5], as shown in Figure 1c. The three-dimensional nanostructures of BPLC are self-assembled and no alignment layer is needed. BPLC achieves fast response time in sub-millisecond range due to the short coherent length. Blue phases including BPI, a body centered cubic structure, BPII, a simple cubic structure, and BPIII, an amorphous structure, can be observed during the supercooling process from the isotropic phase to the chiral nematic [6,7]. SPLCs are composed of three-dimensional twist structures (3-DTSs), where LC directors are twisted from −45° to +45° with respect to the *z* axis on the outermost circumference of the cylinder, with disclinations among them existing between isotropic phase and blue phase in a very narrow temperature range, about several degrees centigrade [8]. The LC molecules in the 3-DTSs of SPLC are depicted in Figure 1d.

**Figure 2:**
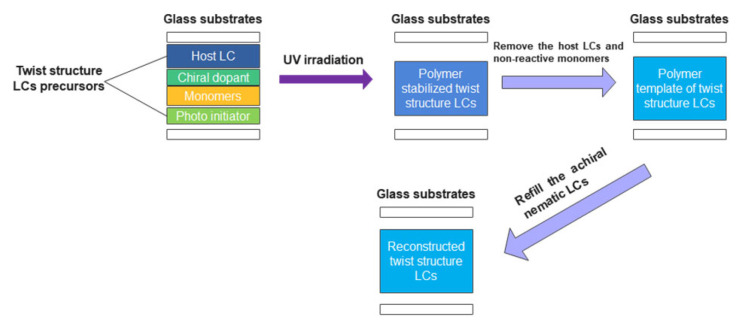
Schematic of templating process for fabricating the TSLCs templates.

Much research has been carried out on the high performance of TSLCs, such as wide bandwidth, high reflectivity, multi-wavelength, and good thermal stability. Among them, templating process is an attractive technique, which may induce the twist structure by polymer template and achiral nematic LCs. In Guo’s work, by refilling a CLC with a right-handed helical structure into the prefabricated polymer network with a left-handed helical structure, a single-layer polymer-stabilized LC film reflecting both right-and left-circularly polarized light was achieved [9]. For BPLC, Castles et al. demonstrated the fabrication of polymer templated blue phase structure [10]. By refilling an achiral nematic LC into a template with chiral three-dimensional structure of the blue phase, the templated BPLC was created that has a temperature range over −125 °C to 125 °C. For SPLC, the templating technique to reconstruct the sphere phase structure was proposed by Chen et al. [11]. With a low concentration polymer template, the thermal stability of SPLCs was improved and the temperature range was broadened to more than 448 K. The polymer template with a helical structure originated from polymer-stabilized TSLCs. The templating process to prepare the polymer template of TSLCs is shown in Figure 2. At first, the precursors of different TSLCs are made respectively by uniformly mixing a nematic LC host, a chiral dopant, monomers, cross-linking agent and a small fraction of photo-initiator on a constant temperature magnetic stirrer. The weight ratios of mixtures with different phases are different. Then, the precursors are capillary into the cells with alignment treatment. Following that, the cells containing the samples are irradiated with ultraviolet light for a certain dosage at different temperatures corresponding to different phases. The polymer network starts to aggregate on the disclination cores of twist structures, stabilizing the cholesteric, blue phase and sphere phase structure. The polymer-stabilized TSLCs are fabricated. After that, the cells were immersed in acetone for about 48 h to remove unpolymerized components including the remaining LC, chiral dopant, crosslinker, and the photo-initiator. Later, the cells were put on the temperature controller to remove the residual ethanol, thus the polymer templates with different phases twist structures were obtained. Then, the polymer templates were refilled with a NLC by a capillary filling process. Thus, the templated-TSLCs (T-TSLCs) were obtained. With the templating technique, TSLCs may show more applications in LC photonic devices.

## 2. Photonic Properties

Among T-TSLCs, cholesteric phase, blue phase and sphere phase are induced by the polymer template, which show the following photonic properties: Bragg reflection, scattering, and optical rotation.

### 2.1. Bragg Reflection and Photonic Band Gap

Due to the self-assembly periodic twist structure of TSLCs, selective reflection of light occurs, a phenomenon called Bragg reflection [12,13,14,15,16]. Bragg reflection consists of wavelength-selectivity and polarization-selectivity. At normal incidence in CLCs, the maximum Bragg reflection wavelength λ_0_ is determined by *p* and *n*, where *p* is pitch length which is mainly determined by chiral dopant concentration and its helical twisting power (HTP), and *n* is the average refractive index ($n=\left(n_{0}+n_{e}\right)/2$, where $n_{0}$ and $n_{e}$ are the ordinary and extraordinary refractive indices, respectively). While in BPLCs, the selective wavelength reflection occurs if the wavelengths are comparable to the lattice constant [17,18]. The maximum Bragg reflection wavelength $\lambda_{B}$ in a BPLC is directly proportional to *n* and *a*, which can be expressed as [2]:

(1) $$\lambda_{B}=\frac{2na}{\sqrt{h^{2}+k^{2}+l^{2}}}$$

where *n* is the average refractive index and *a* is the lattice constant of the blue phase; *h*, *k*, and *l* are Miller indices of various crystal orientation planes. The lattice constant a is the same as the helical pitch length *p* for BPI and is *p*/2 for BPII [2]. Moreover, the Bragg reflection wavelength is also related to the incident angle variation and the bandwidth in BPLC is much narrow than that in CLC [19].

Thus, the reflective color could be tuned by varying the molecular chirality or the concentration of chiral dopant, the pitch *p,* and the optical indices. In addition, the reflective color is also related to external conditions such as temperature, mechanical pressure, electric or magnetic field, angle of incidence of the light, and radiation [20,21,22,23,24,25,26,27]. For an incidence light, high reflection is achieved in a range of wavelengths near the Bragg reflection wavelength, resulting in photonic band gaps (PBGs). The PBG is given by *p* × Δ*n*, and is proportional to the birefringence $\Delta n=n_{e}-n_{0}$. Within the bandwidth, right-circularly polarized light is reflected by a right-handed helix, whereas left-circularly polarized light is transmitted. Outside the bandwidth, both polarization states are transmitted. The bandwidth is currently measured such as the width of the bandgap at half height, which is usually limited to a few tens of nanometers in the visible spectrum because the birefringence is typically limited to 0.5. While the narrow bandwidth is desirable for applications in optical filters, narrow-band polarizers, thermography, and sensors, it also becomes a drawback for innovative applications such as full-color or reflective polarizer-free displays [28,29,30,31,32], broadband polarizers [33,34], and smart windows [35,36,37,38,39].

As linearly polarized light could be regarded as a combination of a left-handed and right-handed circularly polarized component, at Bragg reflection wavelength and normal incidence, one of these components is fully reflected by the twist structure of TSLCs. The other component is transmitted. Therefore, the reflected light is circularly polarized with the same handedness as that of the CLC helical structure and it is valid only at normal incidence, which constitutes the polarization-selectivity [40]. At oblique incidence, the reflected or transmitted light is elliptically polarized. Hence, for unpolarized or linearly incidence light on a TSLC, the reflectance is no more than 50%. However, the reflectivity must be dramatically increased for applications in hyper-reflectivity displays and polarization-independent photonic devices. The fabrication of novel TSLC structure devices overcoming the polarization-selectivity is thus challenging. To overcome the polarization-selectivity, much effort has been accomplished. Ordinarily, the reflectance can exceed 50% when two opposite-handed TSLC films are stacked or two same-handed TSLC films separated by a half waveplate are stacked [41,42,43,44]. However, some issues exist for stacked layers such as the diffusion between layers, optical defects, and losses at the interface. Mitov et al. achieved a single-layer CLC gel from a photopolymerizable monomer that can go beyond the 50% reflectance limit, where the CLC has characteristics of a thermally induced inversion of the helicity sense [45]. The cholesteric gels with two populations of low molar mass LC molecules were also produced to exceed the 50% reflectivity limit [46]. Guo et al. also developed photo- and thermal switching of blue phase liquid crystal films reflecting both right- and left-handed circularly polarized light based on the templating technique [47].

### 2.2. Scattering

Scattering is a phenomenon where the direction of the incident light is changed after striking the obstacles in the medium through which it is traveling. TSLCs can act as scattering particles to generate scattering of light due to the distinct twist structure, which plays a crucial role in random lasing. The CLC possesses helical domains in which the orientation vector successively turns a small torsion angle from one layer to the next one along the helical axis. The helical domains have different refractive indices from the isotropic system, resulting in strong scattering of light [48]. The research focused on the light scattering in CLC with large pitch was reported by Aksenova et al. [49]. They analyzed angular polarization dependencies of the single light scattering intensity as well as the extinction of the mean field. Vernon et al. investigated the mechanism of transforming the CLC into a light-scattering state via the influence of light on the photosensitive alignment layer [50]. While the BPLCs are macroscopic optically isotropic, disordered platelet domain boundaries giving rise to scattering of diffuse light. Scattering also arises from indices mismatch between polymer and LC in polymer-stabilized BPLCs [51]. Sphere phase (SP) is a phase of LC consisting of highly fluid self-assembly of LC molecules 3-DTSs induced by chiral dopant. In SPLC without the electric field, scattering occurs due to the mismatch in the refractive indices of the LC and polymer, which strongly scatters the incidence light in the interface of polymer and LC [52].

### 2.3. Optical Rotation

The helical twisting power in the helical structure of CLCs generates an intense optical rotation, which happens on each side of the reflection band [53]. The Bragg reflection band separates the wavelength regions of optical rotation, and the wavelength regions have opposite signs of rotation. The optical rotation depends strongly on the wavelength of the incidence light. When the light is transmitted along the helical axis, the polarized direction of the incidence light rotates. In BPLC, the nano-scale DTCs arranged in three dimensions give rise to twisting power, leading to a small optical rotatory effect in multiple disordered domains for the incidence light. Thus, the polarization state of the incidence light would be rotated by a small angle [54,55]. The optical rotatory power is mainly proportional to the square of the LC birefringence Δ*n*, and inversely proportional to $\frac{\lambda^{2}}{\lambda_{B}{}^{2}-1}$ where *λ* is the operation wavelength. The rotation angle and optical rotation power increase dramatically when the wavelength gets closer to the Bragg reflection wavelength $\lambda_{B}$, and the optical rotatory power is approximately inversely proportional to $\frac{\lambda^{2}}{\lambda_{B}{}^{2}}$. The optical rotatory power can be positive or negative. For a positive optical rotatory power, the direction of polarization rotation is the right-handed direction that is namely clockwise; for a negative optical rotatory power is left-handed direction that is namely counterclockwise. Liu et al. investigated the polarization rotation of polymer-stabilized BPLCs [56]. They evaluated the effects of birefringence, Bragg reflection wavelength, and incident light wavelength on the optical rotation power of the BPLCs.

## 3. Photonic Applications

T-TSLCs have been employed in widespread photonic applications based on the aforementioned properties. In the following part, we will present the various applications of T-TSLCs, as well as the analysis on the pros and cons of the devices.

### 3.1. Lasing

Due to the helix periodical twist structure, selective Bragg reflection occurs in TSLCs around reflection wavelength, resulting in photonic band gap [57]. Lasing could be excited with its peak wavelength located at the high-energy edge of photonic band gap as long as the gain overcomes the loss because of the band-edge effect [58,59,60].

**Figure 3:**
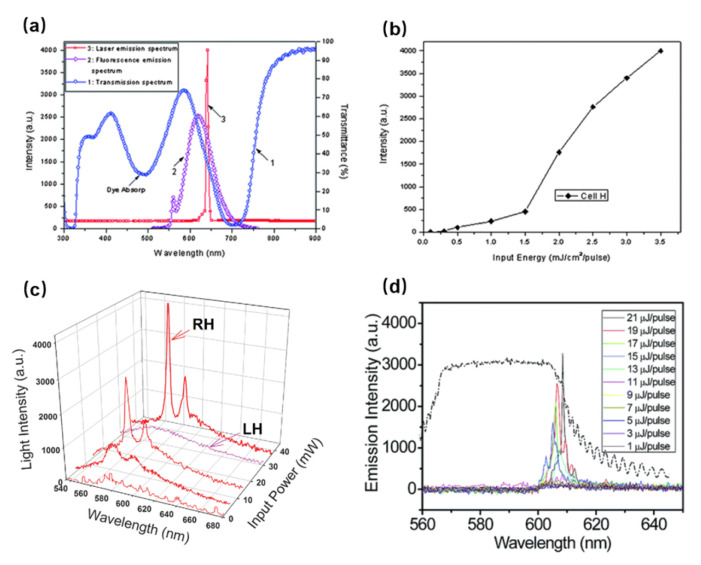
(**a**) Transmission spectrum, fluorescence emission spectrum and laser emission spectrum; (**b**) The dependence of the emission intensity on the excitation light intensity; Reprinted with permission from Ref. [61]. Copyright 2010 Royal Society of Chemistry. (**c**) The laser emission spectrum at different continuous wave (CW) pumping powers. Reprinted with permission from Ref. [62]. Copyright 2012 The Optical Society. (**d**) The fluorescence emission spectrum of the quantum dot CLC polymer template with pumped energy, and corresponding reflection spectra (dashed line). Reprinted with permission from Ref. [63]. Copyright 2014 Royal Society of Chemistry.

The lasing emission with double-handed circularly polarized light based on a CLC template refilled with lasing dye-doped LC was investigated by Guo et al. [61]. The polymer template of CLC with left-handedness originated from a mixture, in which the composition was a non-reactive LC (70 wt% SLC-1717), a diacrylate chiral monomer (10 wt% DCM), a nematic diacrylate monomer (5 wt% C6M), and a chiral dopant (15 wt% S811). The cell containing the polymer template was refilled with samples with right-handed laser dye which was prepared by mixing a non-reactive LC (83.5 wt% SLC-1717), a chiral dopant (16 wt% R811), and an additional laser dye (0.5 wt%). As shown in Figure 3a, the reflection intensity of the dye-doped CLC cell obtained from the polymer template achieves about 100%, that is, both right-handed and left-handed circularly polarized light has been reflected within the same reflection band. A highly efficient lasing condition in comparison with conventional dye-doped CLC lasers was obtained, although only one sharp lasing mode is observed at the high-energy PBG edge, as shown in Figure 3a. The reason is that the reflectance spectrum of PBG deviates from the fluorescent emission band. The emission intensity was significantly enhanced after the light intensity exceeded the pumping energy of 1.5 mJ/pulse, which indicates that the pumping threshold energy for lasing is approximately 1.5 mJ/pulse, as shown in Figure 3b.

The lasing emission device with a double-handed circularly polarized light reflection band can be achieved by utilizing the polymer template of CLC. Moreover, a continuous wave defect-mode lasing of CLC polymer template was realized by Muñoz et al. [62]. They observed the CLC lasing under continuous wave (CW) excitation via a novel structure of the rigid, cross-linked polymer template CLC system with a pitch gradient across the cell thickness. As depicted in Figure 3c, the lasing emission at 572 nm exhibited strong directionality, identical peak emission wavelength, and bandwidth as the laser line observed in the same samples under continuous wave laser excitation at 532 nm. Chen et al. demonstrated the lasing emission in the quantum dot CLCs polymer template [63]. They fabricated a device by surface passivation quantum dots-doped CLC with a polymer template. The lasing emission intensity spectrum of the quantum dot CLCs polymer template and corresponding reflection spectra are shown in Figure 3d. The lasing emission emerged at the band edges of the polymer template, and the threshold was 11 μJ per pulse which was much lower than the quantum dot CLCs band-edge laser without a template. Lin et al. demonstrated gradient-pitched enantiomorphic CLC polymer templates refilled with dye-doped nematic LC, which resulted in lasing emission [64]. In this work, the polymer template is composed of two CLC templates with opposite handedness and the lasing output can distribute from 483.2 nm to 633.7 nm. A thermally convertible laser with an ultra-low lasing threshold based on a gradient-pitched CLC polymer template was also demonstrated [65]. The templated laser has wide-band PBG- tunability in the entire visible region and thermal convertibility between multi-and single-mode band-edge lasing emissions. 

**Figure 4:**
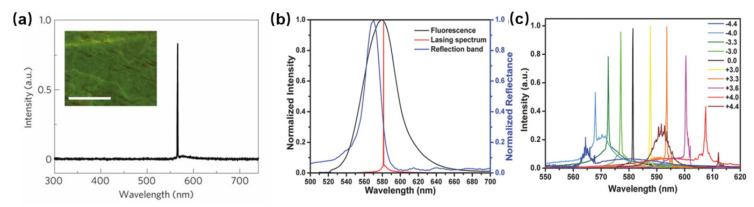
(**a**) Laser emission from the dye-doped BP-templated region; Reprinted with permission from Ref. [10]. Copyright 2012 Springer Nature BV. (**b**) Reflection, fluorescence, and laser emission spectra of the templated BP laser; (**c**) Emission spectra of the templated BP laser at different electric fields. Reprinted with permission from Ref. [68]. Copyright 2018 John Wiley and Sons.

Cao et al. demonstrated lasing emission in BPLC with three-dimensional photonic bandgap structure, but the temperature range was very narrow [66]. Yokoyama et al. realized the lasing emission in a polymer-stabilized BPLC over a temperature range of 35 °C [67]. A method to fabricate self-assembled 3D nanostructure and a laser by polymer templating was proposed by Castles et al., where they used LC of BPI as a template [10]. The fabricated templated blue phases have unprecedented thermal stability in the range of −125 °C to 125 °C. To create a BP-templated laser, the cell with BP template was refilled with a mixture of laser dye. As shown in Figure 4a, the templated blue phase exhibited a clear lasing peak at 565 nm above a threshold input energy, which corresponds to the long-wavelength edge of the template’s bandgap. The results confirm that the lasing emission is a direct result of the polymer template. Wang et al. developed blue phase lasers based on the polymer template doped with fluorescent dyes and observed the lasing emission with electrically tunable wavelength [68]. A templated BP laser was obtained by refilling the laser dye-doped nematic LC, which possesses thermal stability of more than 90 °C and laser emission characteristics with low excitation threshold. As depicted in Figure 4b, a single and narrow emission peak was brought about near the long-wavelength edge PBG by optical excitation with a 532 nm laser beam. The internal distributed feedback of the PBG provided large amplification of the optical gain traveling in the cell due to the long edge overlapping well with maximum fluorescence, obtaining the laser emission with low excitation threshold. Moreover, electric field-dependent shifts of the emission wavelength of the templated BP laser pumped at about 9.5 μJ per pulse are shown in Figure 4c. 

The laser emission occurs in TSLCs based on the band edge effect. Random lasing [69,70], on the other hand, occurs in the TSLCs due to multiple scattering by randomly distributed three kinds of phase platelets. Random lasing has found wide applications in medical diagnostic [71], speckle-free imaging [72], and document coding [73], which arises from multiple scattering and interference effects in a chaotic amplifying medium. In TSLCs, the refractive index mismatch between the twist structure and disclinations gives rise to multiple scattering, and in T-TSLCs, this index mismatch occurs between polymer and LC molecules, resulting in additional light scattering. Coupling of scattering with the gain provided by a laser dye dopant leads to random lasing. 

**Figure 5:**
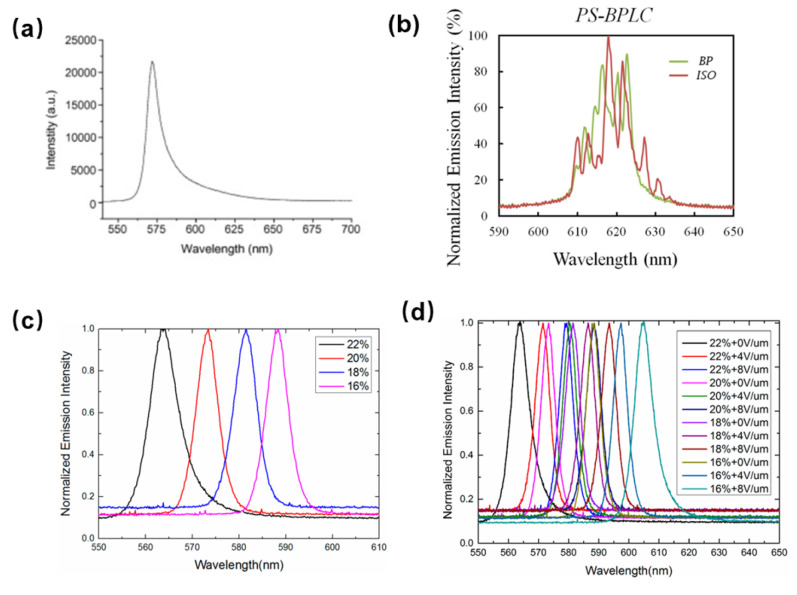
(**a**) Emission spectrum of R6G in 20 wt% (E-CE) C/AA. Reprinted with permission from Ref. [74]. Copyright 2008 Elsevier. (**b**) Emission spectra of PS-BPLC systems in different phases. Reprinted with permission from Ref. [75]. Copyright 2012 The Optical Society. (**c**) Emission spectra of templated SPLC systems with different concentrations of the polymer; (**d**) emission spectra of the templated SPLC systems under different electric fields. Reproduced from Ref. [11]. MDPI, 2017.

He et al. reported a random lasing in a dye-doped CLC polymer solution, in which a mixture of (E-CE) C/AA solution was used as the scatter material, as shown in Figure 5a. They also investigated the effects of concentration of (E-CE) C/AA solution and the thickness of the sample on the random lasing [74]. In BPLC, Chen et al. investigated coherent random lasing from dye-doped BPLCs, in which the random distributed micrometer-size platelets contribute to resonant feedback [75]. In this study, the action of random laser emission occurred in polymer-stabilized BPLC, as illustrated in Figure 5b, where the bandwidth could be tuned by temperature variation. As for the SPLC, Zhu et al. proposed a random lasing occurred by coupling the scattering mechanism with the gain provided by a laser dye dopant, in which the random lasing threshold in the sphere phase was lower than in other mesophases of LCs due to the 3-DTSs structure of SPLC [53]. Moreover, a polymer template is applied to improve the thermal stability and reflection wavelength of random laser. A random lasing with wide temperature and wavelength tunable based on a sphere phase template was proposed by Chen et al. [11]. In their work, the SPLC templates with different polymer concentrations and a mixture of LC and laser dye were prepared. The wavelength tunable random lasing tuned by polymer concentration and electric field was demonstrated, respectively, as shown in Figure 5c,d. The full width at half-maximum (FWHM) is approximately 6 nm and the helical pitch length of the templated SPLC reduces with the increase of polymer concentration, which indicates the wavelength tunable random lasing can be achieved by tuning the polymer concentration. Moreover, the central wavelength of all the samples showed red shift with increasing the electric field, because the director of LC molecule changed with the increasing electric field. Therefore, the central wavelength offset of the templated-SPLC random lasing can be continuously enlarged to 40 nm by the electric field modulation. The random lasing in templated-SPLC exhibits emission spectra with broad bandwidth tuned by electric field variation, which is attractive in the field of random lasing. The investigation in this pioneering research should inspire more new explorations in related areas.

### 3.2. Optical Filters

Optical filters, including Fabry–Perot filters, thin film filters, waveguide filters, and Mach-Zehnder interferometer filters, are one of the crucial components and have been widely applied in various optical systems, especially in optical communications. The unique optical properties of polymer-templated TSLCs can be exploited to provide a wide application in optical filters.

**Figure 6:**
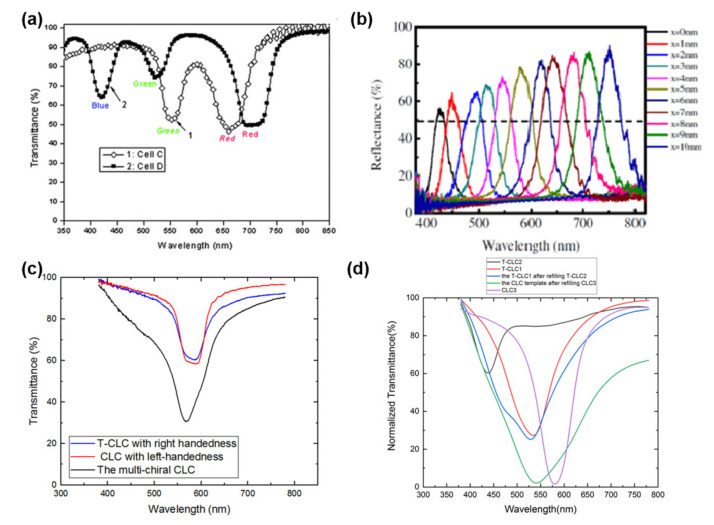
(**a**) The transmission spectra of multiple pitch CLC films; reprinted with permission from Ref. [61]. Copyright 2010 Royal Society of Chemistry. (**b**) Reflection spectra of the spatially-tunable and hyper-reflectivity refilling template sample; reprinted with permission from Ref. [78]. Copyright 2017 SPIE. (**c**) Transmission spectra of high reflectivity CLC filter; Reproduced from Ref. [79]. MDPI, 2021. (**d**) Transmission spectra of wide bandwidth CLC filter. Reproduced from Ref. [80]. MDPI, 2021.

The wavelength and bandwidth of CLC filters can be achieved by tuning thermal or electrical modulation due to the Bragg reflection with a helical structure. Huang et al. demonstrated a bandwidth tunable filter based on the thermal effect on CLCs [76]. The central wavelength can be widely tuned from 826 nm to 517 nm, and the bandwidth can be varied from 10 to 70 nm. Tondiglia et al. reported a broad bandwidth filter with polymer-stabilized CLCs induced by an electric field [77]. However, these methods exhibit some defects such as diffusion, optical losses and defects. A polymer template was used in CLCs to improve the optical properties for the first time by Guo et al., which can be widely applied in optical filters. Guo et al. demonstrated a multi-pitch CLC film with single layer by utilizing the polymer template [61]. In this study, the polymer template was engaged to achieve a simultaneous red, green, and blue reflection band (multiple PBGs) in a single-layer CLC film. As shown in Figure 6a, red/green and red/green/blue colored reflecting CLC films were successfully achieved with the reflection wavelength centered at 662/550 nm and 705/522/423 nm, respectively. The location of the reflection band can be adjusted by changing the concentration of the chiral dopant. This method can extend to a wide region and give rise to new photonic device, where white or multi-color light is manipulated. 

Apart from the multi-pitch CLC film with single-layer based on the polymer template, a wide-band spatially tunable and highly reflective merged CLC template fabricated by combining two templates with two helical structures of opposite handedness was reported by Lin et al. [78]. The fabricated device can simultaneously reflect right- and left-circularly polarized lights and the tunable spectral range includes the entire visible region. As shown in Figure 6b, high reflectance-merged CLC template can be obtained by keeping the temperature over the clearing point of the refilling NLC. The experimental results demonstrate that the maximum reflectance of the device can exceed 85% and the range of wide-band spatial tunability in PBG can be between 400 nm and 800 nm. 

Although Lin et al. proposed a wide-band spatially tunable and highly reflective merged CLC template, there inevitably exist some issues such as complicated construction with the overlapping of two templates and optical loss in the interface. Hence, a high-reflectivity CLC filter with a single-layer template was proposed by Gao et al., which may reflect both right- and left-handed polarized light [79]. The high-reflectivity CLC filters of red, green, and blue color were fabricated by the templating technique, which showed good wavelength consistency. As shown in Figure 6c, the maximal reflectance of the multi-chiral CLC filter is 69% for green color, which has improved by 75% compared to the reflectivity of templated-CLC filter. Moreover, the high-reflectivity of CLC filters for red and blue colors was also achieved. Moreover, a multi-phase LC filter with high reflectance was demonstrated by the single-layer templating technique. A wide-band templated-CLC filter with single-layer based on the multiple wash-out/refill process was proposed by Zhu et al. [80]. The first fabricated CLC template was refilled with a CLC precursor in which the central wavelength differs from the central wavelength of the first CLC template and then was exposed to UV light followed by the wash-out process, thus a second single-layer CLC template that the reflection band was broadened was obtained. As shown in Figure 6d, the CLC filter with wide bandwidth can be fabricated well through the multiple wash-out-refill processes. Moreover, the central wavelength of bandwidth tunable CLC with polymer template is consistent with the intrinsic central wavelength of the CLC template, which verifies the stability of the method. The FWHM of the bandwidth tunable templated-CLC filter can be broadened by 96% compared to the original FWHM of the CLC, and can be continuously broadened with the wide bandwidth covering the entire spectra range from ultraviolet to infrared. Compared with the multi-layer structure of wide bandwidth templated-CLC, the single-layer structure can much simplify the fabrication process.

**Figure 7:**
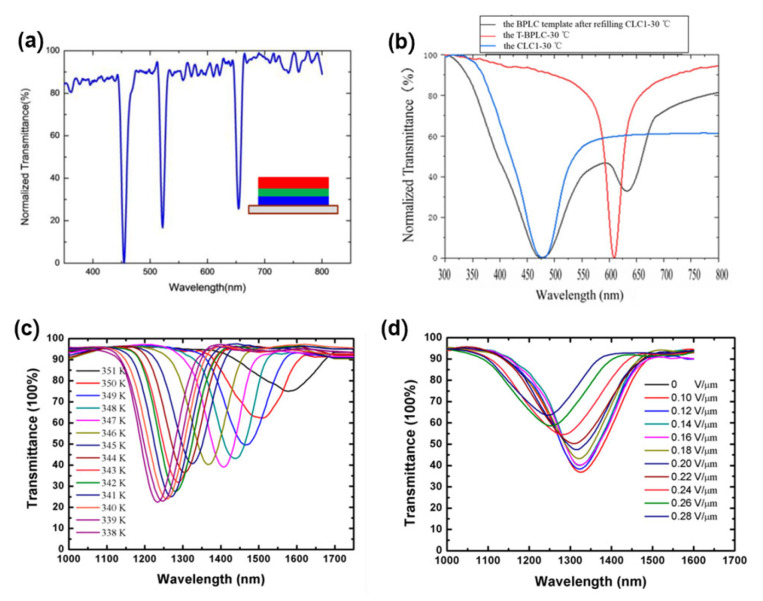
(**a**) The red/green/blue filter with a multi-layer blue phase liquid crystal; reproduced from Ref. [81]. MDPI, 2019. (**b**) Transmission spectra of multi-wavelength LC filter based on the BPLC template; reproduced from Ref. [82]. MDPI, 2021. (**c**) Transmission spectra of the NIR filter at different temperatures; (**d**) electric field dependence of the transmittance on the NIR filter at 345 K. Reproduced from Ref. [83]. MDPI, 2019.

Since the bandwidth of CLC filters is too broad to apply in narrow band-gap filters and the central wavelength of CLC filters will shift after the templating process compared with the templated-BPLC filters, the templated-BPLC with a narrow reflection band is highly desirable to achieve narrow band-gap filters. Zha et al. investigated a multi-wavelength filter in the visible light band based on the BPLC template [81]. In their study, a narrow bandwidth multi-wavelength BPLC device using a polymer template was fabricated, which can realize multi-layer twist structure LC device without the intermediate layer and reflect multiple wavelengths at the same time. As illustrated in Figure 7a, the corresponding wavelengths to reflection peaks of red/green/blue filter are 455, 522, and 654 nm, with the FWHM of 11 nm, 10 nm and 11 nm, respectively. With this method, the multiple reflection peaks determined by the numbers of BPLC templates of the templated-BPLC filters can be achieved and shows good consistency and stability. Whereas the research on multi-wavelength templated-BPLC filters has a potential application in narrow band-gap filters, the design of multi-layer structure was a complication to fabrication process. Therefore, a multi-wavelength templated-BPLC filter with single-layer was proposed, which has advantages of both narrow band-gap filtering characteristic and single-layer structure [82]. In this study, the template effects on the stability of TSLCs were investigated, and a multi-wavelength and a multi-phase TSLC filter would be obtained if refilling a CLC with different pitches into the BPLC template or SPLC template. As depicted in Figure 7b, a dual-wavelength LC filter can be achieved by the wash-out/refill process, which has good stability, consistency, and scalability.

As for SPLC, Sun et al. proposed a near-infrared (NIR) filter with SPLC, which showed a low operating electric field and large temperature-gradient modulations [83]. As shown in Figure 7c, the central wavelength of the sphere phase shifted from 1580 nm to 1324 nm when the temperature decreased from 351 K to 345 K, corresponding to a temperature-gradient of 42.7 nm/K. The central wavelength of the transmittance spectra continuously varied within an electric field of 0.3 V/μm, resulting in a shift of 76 nm (from 1324 nm to 1248 nm) at 345 K, as shown in Figure 7d. Therefore, the fabricated SPLC filter can achieve a wavelength variation of 256 nm by the thermal modulation and 76 nm by electrical modulation, which shows potential applications in optical communication devices.

### 3.3. Gratings

The macroscopic refractive index of LCs is controlled by the electric field, causing the different refractive index at different positions, and the phase accumulated along the optical path of light is modulated. For CLC with self-assembly helical structure, the geometric phase can be endowed into the reflected light within the PBG, while the reflection is highly suppressed outside the PBG. By templating technique, the PBG can be driven reversibly by the electric field. For BPLC exhibiting the Kerr effect, the phase profile can be controlled by non-uniform electric fields or non-uniform Kerr constant distribution. Furthermore, by the templating process, the temperature range can be broadened. For SPLC with sub-millisecond electro-optical switching time under a low switching electric field, the phase can be modulated by the electric field. 

**Figure 8:**
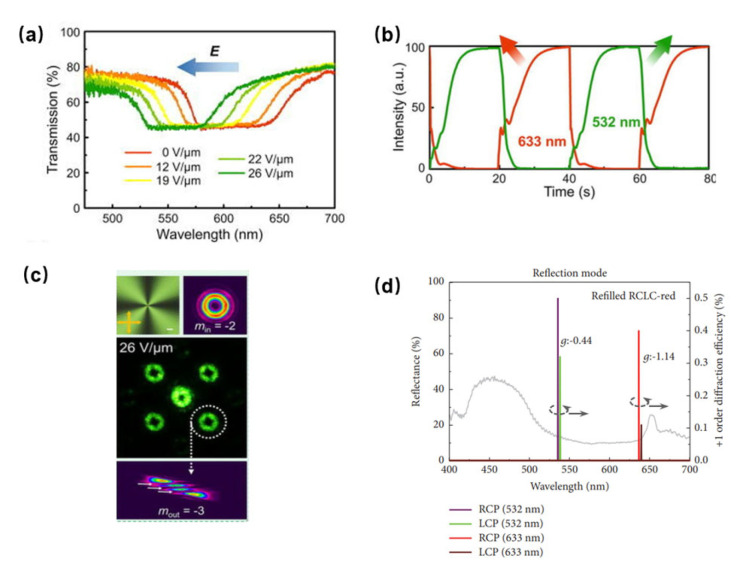
(**a**) Dependency of the PBG shift on applied voltage in the tunable bandpass OV array generator; (**b**) the responsiveness of the tunable bandpass OV array generator; (**c**) POM micrographs of q-plate and the intensity distribution of generated OVs; reprinted with permission from Ref. [86]. Copyright 2021 AIP Publishing. (**d**) Measured +1 order diffraction efficiencies of the refilled CLC template grating. Reproduced from Ref. [87]. Hindawi, 2018.

A grating could be formed when the phase of a TSLC device with polymer template is spatially modulated with a period. Templated CLC can be used to generate the diffraction gratings due to the stabilized planar texture in the polymer template, which is suited to beam steering or sensor protection applications. Subacious et al. described the formation of grating in pure CLC by applying an electric field parallel to the helical axis of a planar-aligned sample [84]. In PS-CLC, Lee et al. used a low concentration polymer network to stabilize electric field-induced diffraction gratings [85]. The stabilized gratings can be electrically switched between a zero-field “on” state, which possesses approximately 75% diffraction efficiency and a moderate field “off” state. For the templated CLC, Hu et al. introduced a dynamic photo-patterning technique to program a templated CLC [86]. A Dammann grating encoded q-plate is fabricated and its function as an optical vortex processor is demonstrated by this method. The Dammann-q-plate (DQP), namely a tunable bandpass optical vortex (OV), is promising in simultaneous orbital angular momentum (OAM) processing, parallel laser fabrication, and micromanipulation. The electro-optical tunability of the bandpass OV array generator was demonstrated by applying the different voltages to polymer templated CLC cells. As illustrated in Figure 8a, as the electric field increases from 0 V/μm to 26 V/μm, the long band edge wavelength shifts from 660 nm to 604 nm, whereas the short band edge wavelength shifts from 570 nm to 523 nm. The result shows that the PBG shift and a continuous color change occur due to the gradual pitch contraction. Moreover, the generated OV shows a high diffraction efficiency and a good energy uniformity by measuring the diffraction efficiency. As vividly depicted in Figure 8b, the response properties are illustrated and the switch on/off times for 633 nm and 532 nm are 10.2 s/1.8 s and 7.8 s/2.9 s, respectively, which enable the dynamic switching of the OV array. Apart from the OV generating, the DQP can work as parallel OAM adders, which can satisfy a broad wavelength range via tuning applied voltage, as shown in Figure 8c. The geometric phase was encoded to a wash-out/refill CLC via a dynamic photo-patterning technique in this study, which is promising in spatial phase modulation of light. On the basis of the band shift, the fast switching of function that is intended for multi-dimensional dynamic control of light is investigated. 

Apart from fabricating a Dammann grating encoded q-plate, the CLC template can be applied to obtain circular polarization and wavelength selective gratings, which was demonstrated by Chin et al. [87]. By refilling left-or right-handed CLC into a right-handed CLC polymer template grating, the refilled CLC template grating can diffract light with a specific wavelength. The refilled CLC template grating shows different diffraction efficiencies for left- and right-handed circular polarization in reflection and transmission modes, as shown in Figure 8d. The fabricated device shows electrical controllability and circular polarization and wavelength selectivity with dual operation modes.

**Figure 9:**
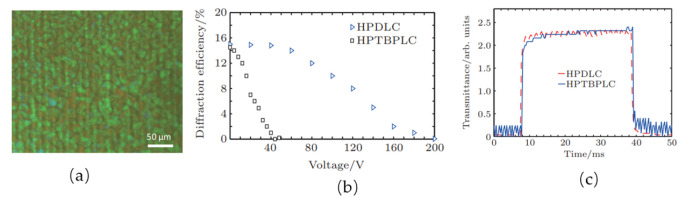
(**a**) Platelet textures observed with the POM for the HPTBPLC grating; (**b**) experimental results for the first-order diffraction efficiencies of HPDLC grating (triangles) and the HPTBPLC grating (squares), respectively; (**c**) measured response times of the HPDLC (dashed curve) and the HPTBPLC (solid curve) gratings. Reprinted with permission from Ref. [88]. Copyright 2015 Chinese Physics B.

To realize a BPLC grating, one can either use patterned electrodes or use periodic polymer slices. For the former, the high resolution is hardly obtained due to the fringing electric field effect, while for the latter, the fabrication is relatively complicated. A holographic polymer templated blue phase liquid crystal (HPTBPLC) grating was accomplished by He et al. [88]. The grating, obtained by a periodic fringe polymer template refilled with BPLC, can remarkably reduce its switching voltage from 200 V to 43 V while maintaining a sub-millisecond response time. The morphology of the HPTBPLC grating is depicted in Figure 9a, where the self-assembled BPLC shows a characteristic platelet texture based on the polymer template. The different colors originate from different orientations of cubic lattices. As illustrated in Figure 9b, the switching voltage of HPDLC grating is 200 V, while the switching voltage of HPTBPLC is 43 V, which has a significant reduction. The HPTBPLC could maintain a sub-millisecond response time shown in Figure 9c, resulting from the fast response time of BPLC. Usually, according to the Gerber model, a BPLC can exhibit a sub-millisecond property if the concentration of chiral dopant is appropriate. The response time of the HPTBPLC could be improved by increasing chiral dopant concentration.

**Figure 10:**
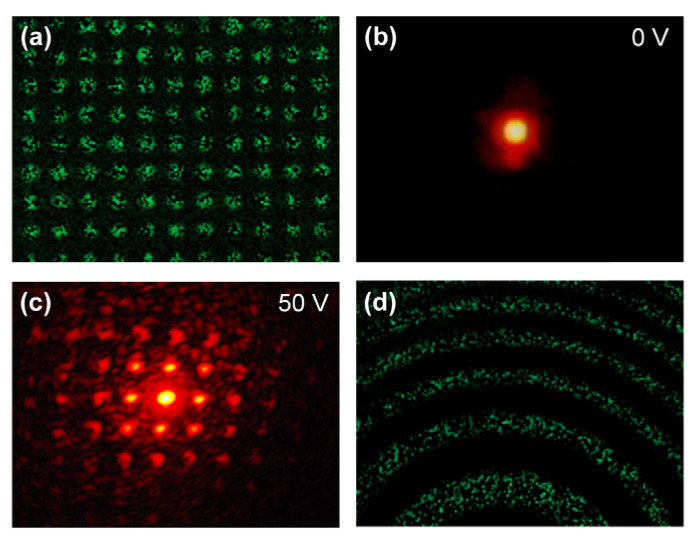
(**a**) Reflection micrograph; (**b**,**c**) far field diffraction patterns of a 2D BP-ISO array obtained by erasing the previous 1D grating and repatterning the sample into a 2D grating; (**d**) reflection micrograph of the same blue phase templated azo-LC cell repatterned into a Fresnel zone plate. Reproduced from Ref. [89]. Optical Society of America, 2019.

Jau et al. proposed an optically rewritable dynamic phase grating based on polymer templated azo LC in a blue phase structure [89]. The refractive index in the proposed grating can be spatially modulated without patterned electrodes. The dynamic grating includes blue phase and photo-induced isotropic-phase (ISO) regions, where the index changes in the blue-phase regions under external voltage and remains unchanged in the ISO regions. The grating shows significant characteristics of polarization independence and sub-millisecond electro-optic response. To characterize the optical rewritable of the blue phase templated azo-LC cell, the authors accomplished the following experiment. At first, with green light erasing the pattern, the 1D phase grating is irradiated to reconfigure the ISO region back to BP region. Then, a 2D BP-ISO array, using a 2D grating photomask, is obtained by UV irradiation shown in Figure 10a. No diffraction is observed without applied voltage shown in Figure 10b, while a 2D array appears with increasing the field to 50 V shown in Figure 10c. Later, the 2D grating pattern is erased and the sample is rewritten into a Fresnel zone plate, which exhibits well electric-optic characteristics, as shown in Figure 10d. 

The SPLC is promising for obtaining grating due to the 3-DTSs structure and unstable disclinations among them. Li et al. demonstrated a fast switchable dual-model grating based on a polymer-stabilized SPLC [90]. Both the phase and the amplitude can be modulated by the fabricated grating, in which diffraction efficiency was six times of grating fabricated with polymer-stabilized BPLC. In addition, the grating possesses polarization-independent and sub-millisecond response time, which exhibits great potential for diffractive optics.

## 4. Other Applications

TSLCs can also be applied in some fields such as nanomaterials, structured light, and biomedicine. Hu et al. investigated the reconstruction capability of the BPLC template with low polymer concentration [91]. They confirmed a threshold polymer concentration by using different polymer systems to reconstruct the blue phase with the chiral three-dimensional template. In this paper, three kinds of the material systems were prepared to obtain the blue phase template, in which the mixture included a positive NLC (BPH006), a chiral dopant (R5011), a cross-linking agent (C3M), an ultraviolet curable monomer (TMPTA, EHA, and 12A), and a photoinitiator (IRG184). The experimental results show that the threshold polymer concentrations for the blue phase reconstruction with monomer TMPTA, EHA, and 12A are confirmed to be 10 wt%, 14 wt%, and 16 wt%, respectively. Therefore, for different polymer materials reconstructing the blue phase template, the threshold values of polymer concentration are different. The refilling effect was also investigated by refilling the different LCs. The reverse-handed chiral LCs acting as refilling materials could improve the driving voltage. In the field of structured light, an important example, of fundamental and practical interest, has been light beams structured to carry OAM. The T-TSLCs can be used to manipulate light beams with OAM. Zhu et al. proposed an innovative scheme for the simultaneous manipulation of the Pancharatnam-Berry (PB) phase via coexisting two CLCs with opposite chirality [92]. The system was demonstrated by refilling CLC into a polymer template with opposite chirality, which can reflect both circularly polarized light. Accordingly, the reflective OV with opposite topological charge was generated. Compared to traditional CLC stacks or mirror-backed CLC devices, the proposed method shows important merits of ultra-compact device configuration and higher efficiency, which facilitates the architectures and functionalities of structured light in TSLCs toward photonic devices. The CLC can also be studied in biomedicine due to the helical structure. Lee et al. proposed a detection method by observing the coloring pattern of CLC droplets, which can be used as biosensors [93]. In this paper, the helical structures and reflecting color patterns of high- and low-dopant CLC droplets coated with poly (vinyl alcohol) (PVA) and sodium dodecyl sulfate (SDS) were studied. The produced CLC droplets show high sensitivity, good selectivity, and fast response. 

## 5. Conclusions

T-TSLCs possess attractive characteristics: strong thermal stability, fast response, low driving voltage, and simple fabrication. Herein, TSLCs with polymer templates have been employed for various photonic applications such as lasing, optical filters, and gratings. The configuration, fabrication process, and photonic properties of T-TSLC devices have been reviewed. However, there are still challenges for T-TSLCs remaining to be overcome. Due to the need to remove the LC during the templating process and to determine if the LC has been fully removed, the production costs will be increased by invoking additional manipulations. The scalability of the production is still a limitation because the polymer template is produced in little LC cells. The research on T-TSLCs is fascinating and the potential of the templated structures in nature is massive. In order to form complex geometries in 2D and 3D processes for advance applications, the templated structures will be required to be integrated into functional devices. Moreover, the dynamic capabilities of the polymer templates remain to be explored. With the developments of materials and device structures, the T-TSLCs hold significant potential for photonic applications. 

## Data Availability

Our study did not report any data.
